# Predicting intraventricular hemorrhage growth with a machine learning-based, radiomics-clinical model

**DOI:** 10.18632/aging.202954

**Published:** 2021-05-04

**Authors:** Dong-Qin Zhu, Qian Chen, Yi-Lan Xiang, Chen-Yi Zhan, Ming-Yue Zhang, Chao Chen, Qi-Chuan Zhuge, Wei-Jian Chen, Xiao-Ming Yang, Yun-Jun Yang

**Affiliations:** 1 Department of Radiology, The First Affiliated Hospital of Wenzhou Medical University, Wenzhou 325000, China; 2 Department of Radiology, Lab-Yang, University of Washington, Seattle, WA 98109, USA; 3 Department of Neurosurgery, The First Affiliated Hospital of Wenzhou Medical University, Wenzhou 325000, China

**Keywords:** cerebral intraventricular hemorrhage, machine learning, decision support techniques, precision medicine, multidetector computed tomography

## Abstract

We constructed a radiomics-clinical model to predict intraventricular hemorrhage (IVH) growth after spontaneous intracerebral hematoma. The model was developed using a training cohort (N=626) and validated with an independent testing cohort (N=270). Radiomics features and clinical predictors were selected using the least absolute shrinkage and selection operator (LASSO) method and multivariate analysis. The radiomics score (Rad-score) was calculated through linear combination of selected features multiplied by their respective LASSO coefficients. The support vector machine (SVM) method was used to construct the model. IVH growth was experienced by 13.4% and 13.7% of patients in the training and testing cohorts, respectively. The Rad-score was associated with severe IVH and poor outcome. Independent predictors of IVH growth included hypercholesterolemia (odds ratio [OR], 0.12 [95%CI, 0.02-0.90]; p=0.039), baseline Graeb score (OR, 1.26 [95%CI, 1.16-1.36]; p<0.001), time to initial CT (OR, 0.70 [95%CI, 0.58-0.86]; p<0.001), international normalized ratio (OR, 4.27 [95%CI, 1.40, 13.0]; p=0.011), and Rad-score (OR, 2.3 [95%CI, 1.6-3.3]; p<0.001). In the training cohort, the model achieved an AUC of 0.78, sensitivity of 0.83, and specificity of 0.66. In the testing cohort, AUC, sensitivity, and specificity were 0.71, 0.81, and 0.64, respectively. This radiomics-clinical model thus has the potential to predict IVH growth.

## INTRODUCTION

Intraventricular hemorrhage (IVH) occurs in up to 54% of intracerebral hemorrhage (ICH) cases [1], and is considered an independent predictor of poor outcome due to the mass effect, obstructive hydrocephalus, and inflammatory meningitis [2–5]. Twelve percent of IVH cases will increase in volume by more than 2 mL.

Radiologists distinguish growth-prone hematoma based on empirical knowledge of location, radiological sign, and morphological characteristics. However, these qualitative variables are subjective and difficult to standardize. Furthermore, atypical hematomas with fine borders can develop new IVH upon follow-up examinations. These characteristics hamper diagnosis and treatment decisions.

Radiomics is an emerging tool that allows researchers to obtain quantitative features from medical images. Radiomics features can be used to evaluate the tumor spatial heterogeneity and microenvironment and reflect tumor gene patterns [6]. It is widely used for evaluating tumor prognosis, selecting appropriate treatment, and predicting lymph node metastasis [7, 8]. Although some researchers have predicted parenchymal hemorrhage enlargement with radiomics technology [9–11], few have tried to predict IVH growth. In this study, we aimed to develop a model that incorporates clinical and radiomics features to identify patients at high risk for IVH growth in the acute phase of ICH.

## RESULTS

### Study population

The incidence of IVH growth in the training and testing cohorts were 84 (13.4%) and 37 (13.7%), respectively (Table 1). There was no significant difference in the incidence of IVH growth or baseline characteristics between the cohorts.

**Table 1:** Baseline characteristics of patients in the training and testing cohorts (variables were presented as counts [percentages]).

**Training cohort (N=626)**			**Testing cohort (N=270)**		
Baseline IVH (%)*	IVH expansion	51.0 (24.2)	Baseline IVH (%)*	IVH expansion	20 (20.0)
	Non-IVH expansion	160.0 (75.8)		Non-IVH expansion	79 (80.0)
No baseline IVH (%)*	New IVH	33.0 (8.0)	No baseline IVH (%)*	New IVH	17 (9.9)
	No IVH	382.0 (92.0)		No IVH	154 (90.1)
IVH growth (%)*		84.0 (13.4)	IVH growth (%)*		37.0 (13.7)

IVH= intraventricular hemorrhage; *Data are the number of patients, with percentages in parentheses.

### Patient characteristics

Among the 626 patients enrolled in the training cohort, those with IVH growth had a lower admission GCS (median [IQR], 12.5 [7.0-15.0] vs. 14.0 [11.0-15.0]; p<0.001), larger parenchymal hemorrhage volume (median [IQR], 19.6 [11.8-30.5] mL vs. 15.9 [9.4-26.0] mL; p<0.01), higher baseline Graeb score (3.0 [0.0, 7.8] vs. 0.0 [0.0. 2.0]; p<0.001), and experienced a shorter time from onset to initial ICH detection (median [IQR], 2.0 [1.5-3.5] h vs. 3.0 [2.0-4.5] h; p<0.01) (Table 2). Patients without a history of hypercholesterolemia (1.0 [1.2%] vs. 76.0 [14.0%)]; p<0.01) or hypertension (62.0 [73.8%] vs. 451.0 [83.2%]; p<0.05) were more likely to experience IVH growth. Patients with IVH growth were more likely to have parenchymal hemorrhage expansion (HE) (47.0 [56.0%] vs. 80.0 [14.8%]; p<0.001) and a poor outcome (81.0 [96.4%] vs. 405.0 [74.7%]; p<0.01). GCS, baseline ICH volume, baseline Graeb score, time to initial CT, blood glucose, history of hypercholesterolemia, hypertension, international normalized ratio (INR), and the Rad-score were analyzed by multivariable regression.

**Table 2:** Characteristics of patients in two cohorts and univariate analysis of variables associated with IVH growth.

	**Training cohort (N=626)**			**Testing cohort (N=270)**		
	IVH growth	Non-IVH growth	P value	IVH growth	Non-IVH growth	P value
**Age (year)**	61.5(53.0, 73.8)	60.0(51.0, 69.0)	0.285	65.0(55.0, 68.5)	59.0(50.0, 67.0)	0.045
**Sex**			0.198			0.927
Male (%)*	61.0(72.6%)	355.0(65.5%)		23.0(62.2%)	143.0(61.4%)	
Female (%)*	23.0(27.4%)	187.0(34.5%)		14.0(37.8%)	90.0(38.6%)	
Hypertension (%)*	62.0(73.8%)	451.0(83.2%)	0.037	25.0(67.6%)	192.0(82.4%)	0.035
Hypercholesterolemia (%)*	1.0(1.2%)	76.0(14%)	0.001	3.0(8.1%)	23.0(9.9%)	0.970
Diabetes (%)*	5.0(6.0%)	53.0(9.8%)	0.260	5.0(13.5%)	30.0(12.9%)	1.000
Prior hemorrhage (%)*	4.0(4.8%)	20.0(3.7%)	0.864	2.0(5.4%)	9.0(3.9%)	1.000
Alcohol-consumption (%)*	25(29.8%)	161(29.7%)	0.991	10.0(27.0%)	61.0(26.2%)	0.913
**GCS**	12.5(7.0, 15.0)	14.0(11.0, 15.0)	<0.001	10.0(7.0, 13.0)	13.0(10.0, 15.0)	<0.001
**Time to initial CT (h)**	2.0(1.5, 3.5)	3.0(2.0, 4.5)	0.001	2.5(2.0, 3.5)	3.0(2.0, 4.0)	0.474
**Baseline ICH volume (mL)**	19.6(11.8, 30.5)	15.9(9.4, 26.0)	0.008	21.1(12.8, 38.0)	17.0(9.0, 27.5)	0.009
Parenchymal HE (%)*	47.0(56.0%)	80.0(14.8%)	<0.001	20.0(54.1%)	35.0(15.0%)	<0.001
**Blood glucose (mmol/L)**	7.4(6.2, 9.0)	6.7(5.9, 8.0)	0.009	7.2(6.2, 9.2)	6.9(6.0, 8.3)	0.177
**PLT count (10^9/L)**	195.5(159.5, 226.8)	206.0 (169.8, 245.3)	0.051	204.0(143.0, 228.0)	201.0(163.0, 246.0)	0.219
**INR**	1.0(1.0, 1.1)	1.0(1.0, 1.1)	0.087	1.0(1.0, 1.1)	1.0(1.0, 1.0)	0.666
**APTT (s)**	34.2(31.4, 37.8)	34.0(31.1, 37.2)	0.492	32.9(31.2, 36.0)	33.3(31.2, 36.8)	0.825
**Baseline Graeb score**	3.0(0.0, 7.8)	0.0(0.0, 2.0)	<0.001	2.0(0.0, 5.0)	0.0(0.0, 2.0)	0.012
**Follow-up Graeb score**	5.0(3.0, 8.0)	0.0(0.0, 2.0)	<0.001	4.0(3.0, 6.5)	0.0(0.0, 2.5)	<0.001
GOS≤3 (%)*	81.0(96.4%)	405(74.7%)	<0.001	35.0(94.6%)	169.0(72.5%)	0.004
**Rad-score**	-1.5(-2.0, -1.1)	-2.1(-2.6, -1.6)	<0.001	-1.7(-2.0, -1.2)	-2.1(-2.7, -1.7)	0.001

CT= computed tomography; GCS= Glasgow Coma Scale; GOS= Glasgow Outcome Scale; ICH= intracerebral hemorrhage; HE= hemorrhage expansion; IVH= intraventricular hemorrhage; PLT= platelet; INR= international normalized ratio; APTT= activated partial thromboplastin time; Rad-score= radiomics score.

Continuous variables were presented as medians (interquartile range, [IQR]) and categorical variables were presented as counts (with percentages). P values were calculated by using χ2 test or Wilcoxon rank-sum test. *Data are the number of patients, with percentages in parentheses.

Multivariable regression analysis indicated that a history of hypercholesterolemia (odds ratio [OR], 0.12 [95% CI, 0.02-0.90]; p=0.039), baseline Graeb score (OR, 1.26 [95% CI, 1.16-1.36]; p<0.001), time to initial CT (OR, 0.70 [95% CI, 0.58-0.86]; p<0.001), INR (OR, 4.27 [95% CI, 1.40, 13.00]; p=0.011), and Rad-score (OR, 2.30 [95% CI, 1.60-3.30]; p<0.001) (Supplementary Table 2) were independently associated with IVH growth. Multicollinearity was not observed between the independent predictors and IVH growth (VIF for all <2).

### Radiomics analysis

**Figure 1:**
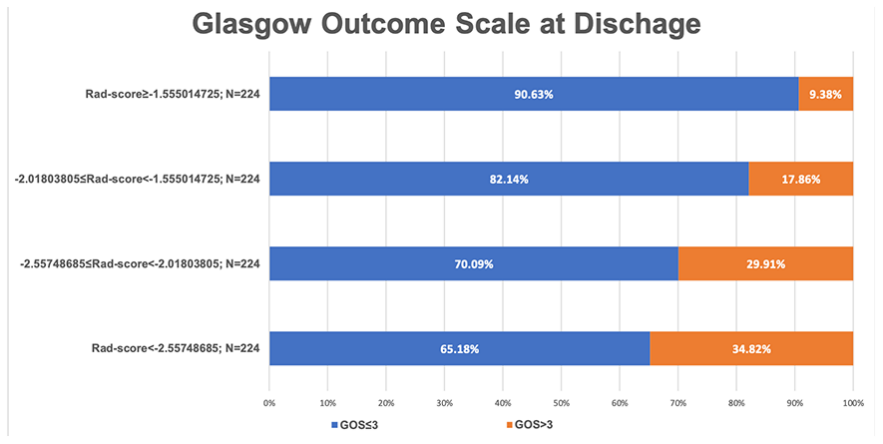
A bar chart demonstrating the relationship between the Rad-score and glasgow outcome scale at discharge.

The median ICC of 396 candidate features was 0.96 (IQR, 0.87-0.99). Twenty-eight features were excluded and 368 features with good agreement were further analyzed by the LASSO regression model. Finally, seven features were selected to construct the radiomics signature (Supplementary Figure 1, Supplementary Table 3). The mean and median ICC of the seven selected features were 0.88 (standard deviation, 0.06) and 0.90 (IQR, 0.85-0.91), respectively. The Rad-score was calculated through the linear combination of selected features multiplied by their respective LASSO coefficients (Supplementary Material 3). The Rad-score was confirmed to be a significant predictor of IVH growth with an optimal cut-off value of -1.7259179. Patients with a Rad-score of ≥-1.7259179 were more likely to encounter severe IVH (Graeb score, ≥6, 50.0 [22.6%] vs. 55.0 [13.6%]; p=0.004) and have a poor outcome (GOS, ≤3, 197.0 [89.1%] vs. 289.0 [71.4%]; p<0.001) (Table 3). The results from Figure 1 indicate that the proportion of poor outcomes was lower in individuals with a lower Rad-score and progressively increased with an increasing interquartile Rad-score (p<0.001). We used the Rad-score to predict poor outcome. In the training cohort, the Rad-score achieved an AUC of 0.695, a sensitivity of 0.639, and a specificity of 0.696. In the testing cohort, the AUC, sensitivity, and specificity were 0.665, 0.639, and 0.632, respectively. The Receiver-operator curves are shown in Supplementary Figure 2.

**Table 3:** Relationship between Rad-score, severe IVH and poor outcome at discharge. (Variables were presented as counts [percentages]).

**N (%)**	**Severe IVH (Greab score, ≥6)**	**Poor outcome (GOS, ≤3)**
Rad-score≥-1.7259179 (%)*	50.0(22.6)	197.0(89.1)
Rad-score<-1.7259179 (%)*	55.0(13.6)	289.0(71.4)
**P value**	0.004	<0.001

P values were calculated by using χ2 test. *Data are the number of patients, with percentages in parentheses.

### Model performance

**Figure 2:**
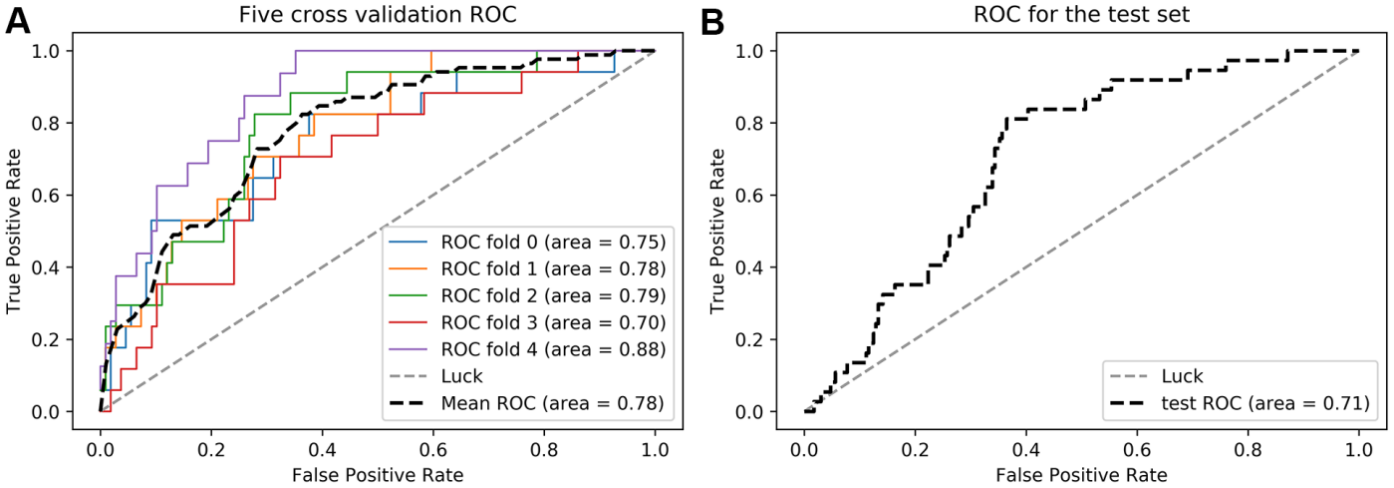
Receiver operator curves (ROC) of the radiomics-clinical model in the training (**A**) and testing (**B**) cohorts.

Five features, including history of hypercholesterolemia, baseline Graeb score, time to initial CT, INR, and Rad-score, were introduced into the SVM model. In the training cohort, the model yielded an AUC of 0.78, sensitivity of 0.83, and specificity of 0.66. In the testing cohort, the AUC, sensitivity, and specificity were 0.71, 0.81, and 0.64, respectively Figure 2, Table 4. The confusion matrices results of the model are shown in Supplementary Tables 4, 5.

**Table 4:** Performance of the radiomics-clinical model.

	**ACC**	**AUC**	**Sensitivity**	**Specificity**	**PPV**	**NPV**
**Training cohort**	0.75	0.78	0.83	0.66	0.27	0.96
**Testing cohort**	0.71	0.71	0.81	0.64	0.26	0.95

ACC=area under the receiver operating curve; ACC=accuracy; PPV=positive predictive value; NPV=negative predictive value.

### Evaluation of clinical usefulness

**Figure 3:**
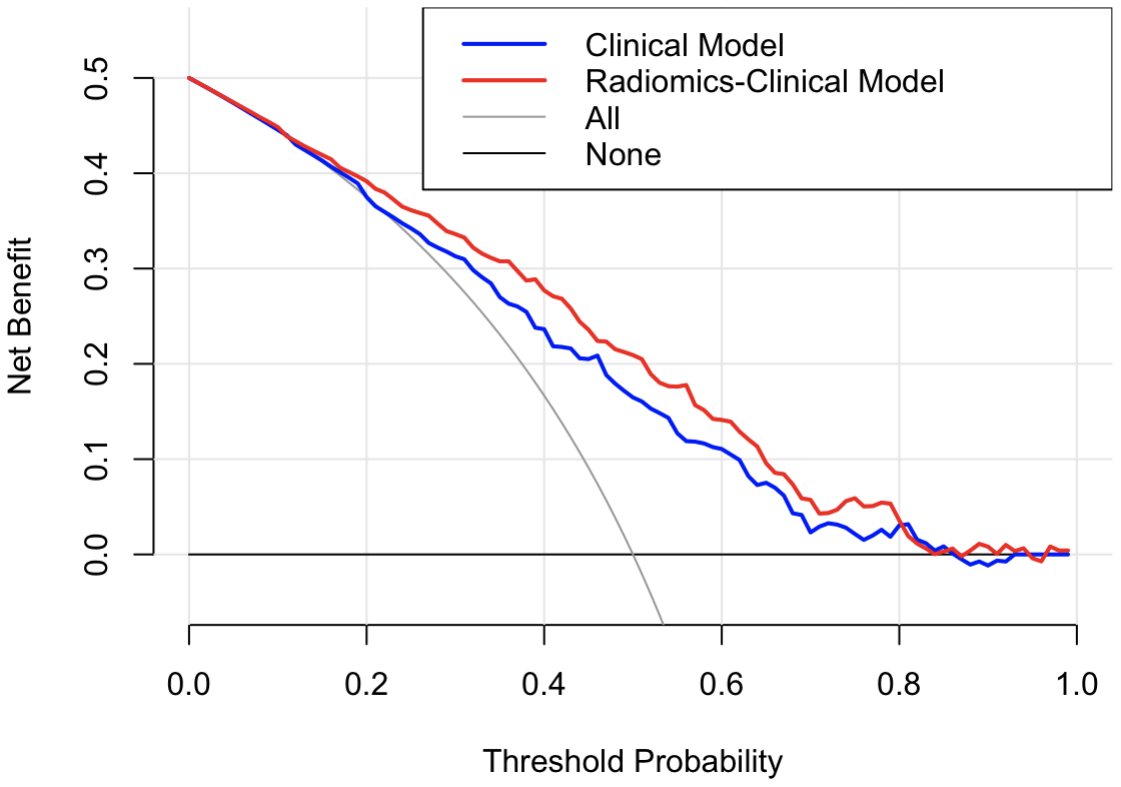
**Decision curve analysis for the two models.** The y-axis indicates the net benefit; the x-axis indicates threshold probability. The grey line represents the assumption that all patients have IVH growth. The black line represents the assumption that no patients have IVH growth. The blue line and red line represent the net benefit of the single clinical model and radiomics-clinical model, respectively. The radiomics-clinical model had a higher net benefit compared with the single clinical model across most threshold probabilities $\text{Net benefit}={\text{TPR}}_{\text{R}}\text{ P}-\frac{\text{R}}{1-R}\;FPR_{\text{R}}\;(1-P),$ P represents the prevalence of the disease; R represents the threshold probability; TPR= true positive rate; FPR= false positive rate).

DCA indicated that the radiomics-clinical model had a higher overall net benefit in distinguishing patients at high risk for IVH growth than the single clinical model for most of the threshold probabilities (Figure 3).

## DISCUSSION

A radiomics-clinical model predicting IVH growth was established using SVM and showed good performance. The Rad-score was confirmed to be independently associated with severe IVH and poor outcomes. To the best of our knowledge, this is the first application of radiomics to predict IVH growth in a relatively large sample (a total of 896 patients enrolled).

**Figure 4:**
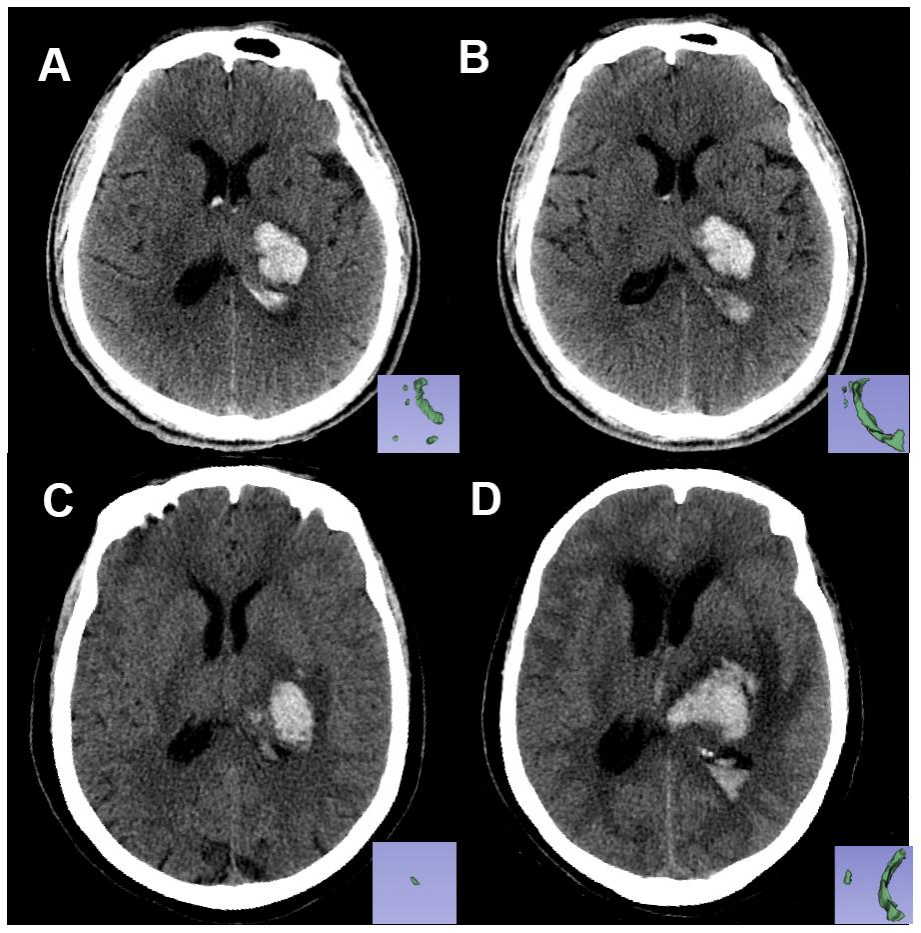
**Non-contrast CT images of two patients with similar hematoma but different experiences.** Pictures in the lower right corner are the 3-D images of the IVH. Image (**A**, **B**) are the baseline and follow-up CT images, respectively, of patient A: a 61-year-old male who had a Rad-score of -2.2119681 (<-1.7259179). Image (**C**, **D**) are the baseline and follow-up CT images, respectively, of patient B: a 67-year-old female with a Rad-score of -1.6176548 (>-1.7259179). Within 24 hours from symptom onset, the IVH volume of patient A changed from 5.62 mL to 6.84 mL, and the IVH volume of patient B changed from 0.5 mL to 12.4 mL. Patient B experienced IVH growth while patient A did not.

Among the seven radiomics features used to construct the Rad-score, features from group GLZSM characterized the texture homogeneity of lesions, and the Feature Haralick Correlation from group GLCM measured the degree of image gray level similarity. Previous reports indicated that hematoma heterogeneity was a sign of active bleeding and could predict hematoma development [10, 12]. We inferred that the radiomics features captured the intrahematomal heterogeneity. Nevertheless, interpreting the association between the radiomics features and the underlying biological processes is challenging. The Rad-score incorporates multiple radiomics features and serves as a multi-factor panel that reduces the complexity of multi-feature studies [13]. For example, the Rad-score could differentiate between similar hematomas in two patients and specify their different outcomes (Figure 4).

Our study also found that increasing INR levels and baseline Graeb scores are predictors for IVH growth. A higher INR implies longer prothrombin time and worse coagulation function, which could cause bleeding and lead to further IVH growth, as seen in both ventricular [14] and parenchymal hemorrhage growth [15–18]. The Graeb score is a grading system of 0–12, which can be used to quantify the amount of blood in each ventricle [19]. Increases in the Graeb score indicates a larger ventricular hemorrhage and can be used to predict IVH growth.

A history of hypercholesterolemia was associated with a lower risk of IVH growth in our study. Higher serum cholesterol is negatively correlated to ICH volume and HE risk in parenchymal hematoma [20, 21]. There is also a protective association between hypercholesterolemia and ICH risk (especially in non-lobar lesions) [22–24], regardless of whether the patient uses statins [20, 22]. These correlations might be explained by the role of serum cholesterol in maintaining vascular integrity and promoting platelet aggregability [21]. Moreover, a higher level of triglycerides is related to a lower rate of deep microbleeds [25]. Thus, hypercholesterolemia may contribute to the maintenance of deep penetrating arterioles [22] and prevent IVH growth.

An increasing number of studies have focused on the contribution of IVH severity to prognosis and the tools for grading IVH extent [1, 26, 27]. A Graeb score of ≥5 was reported as an independent predictor of poor outcome [1]. External ventricular drain (EVD) placement or prior treatment was recommended for patients with a Graeb score of >5 [28]. Thus, we defined patients with a follow-up Graeb score of ≥6 as having severe IVH. Our results showed that the Rad-score could predict IVH growth, and distinguish patients who might develop severe IVH (Graeb score, ≥6) or have poor outcomes (GOS, ≤3) at the first CT scan.

Currently, IVH management involves EVD, intraventricular fibrinolysis, neuro-endoscopic procedure, and lumbar drainage [29–31]. However, there is no precise clinical threshold that determines the need for EVD or thrombolysis [15]. Furthermore, there are complications regardless of the method chosen, and the net benefit of these invasive therapies remains unclear [5]. Some studies reported no significant difference in the outcome of patients who received two different treatments [32]. This emphasizes the need for early identification of patients at high risks for IVH growth and offering them targeted treatment when the rate of hematoma growth is highest [2, 5].

The radiomics-clinical model in our study can identify the high-risk patient group by a noninvasive method. Accordingly, this can help clinicians judge the patient’s condition and select a treatment.

There were several limitations in our study. First, this is a retrospective study. The time for repeating the CT scan is varied, which may underestimate the extent of IVH expansion and hydrocephalus. Second, the single-center enrollment is limited in its generalizability. Hence, a future prospective and multi-institutional study is needed. Third, it takes approximately two to five minutes to segment a complete ROI. This manual delineation of hematomas is time-consuming and has a relatively low reproducibility. Consequently, we are exploring the feasibility of an automated or semi-automated delineation of hematoma. Finally, a threshold of 2 mL may exclude patients with a volume increase of <2 mL but who still developed severe IVH. For instance, a previous study found an expansion of 1 mL in IVH volume was associated with poor outcomes [3]. Therefore, additional studies are required to further confirm the cut-off value that correlates to poor outcome.

In conclusion, we confirmed that the Rad-score at admission was associated with severe IVH and poor outcome. Our model incorporates radiomics and clinical variables and was developed using the SVM method. The radiomics-clinical model predicted IVH growth with good performance and may help clinicians target patients who have a high IVH growth risk.

## MATERIALS AND METHODS

Our study was approved by the Medical Ethics Committee of The First Affiliated Hospital of Wenzhou Medical University and written informed consent was waived.

### Patients and clinical data

Patients with ICH seen between September 2013 and August 2018 in The First Affiliated Hospital of Wenzhou Medical University were retrospectively reviewed. Those who were >18 years old and received baseline and follow-up CT scans within 6 h and 72 h from the onset were included. Exclusion criteria were as follows: (1) secondary ICH caused by aneurysm, arteriovenous malformation, neoplasm, hemorrhagic infarction, or traumatic brain injury; (2) surgery or interventional therapy before follow-up CT scan; (3) primary IVH; (4) non-deep ICH location (lobar, cerebellum or brain stem hematoma); (5) use of anticoagulants or antiplatelet drugs before ICH onset; and (6) CT images with severe artifacts. Finally, a total of 896 patients were included and randomly divided into the training (N=626) and testing (N=270) cohorts.

Demographic data (age and sex), medical history (history of hypertension, diabetes, hypercholesterolemia, hemorrhage, and alcohol consumption), initial clinical data (Glasgow coma scale, time to initial CT, and baseline ICH volume), and laboratory data (blood glucose, platelet count, international normalized ratio, and activated partial thromboplastin time) were recorded after admission. The Glasgow Outcome Scale (GOS) was evaluated at discharge, and poor outcome was defined as having a GOS of ≤3 [33, 34].

### Neuroimage acquisition and analysis

**Figure 5:**
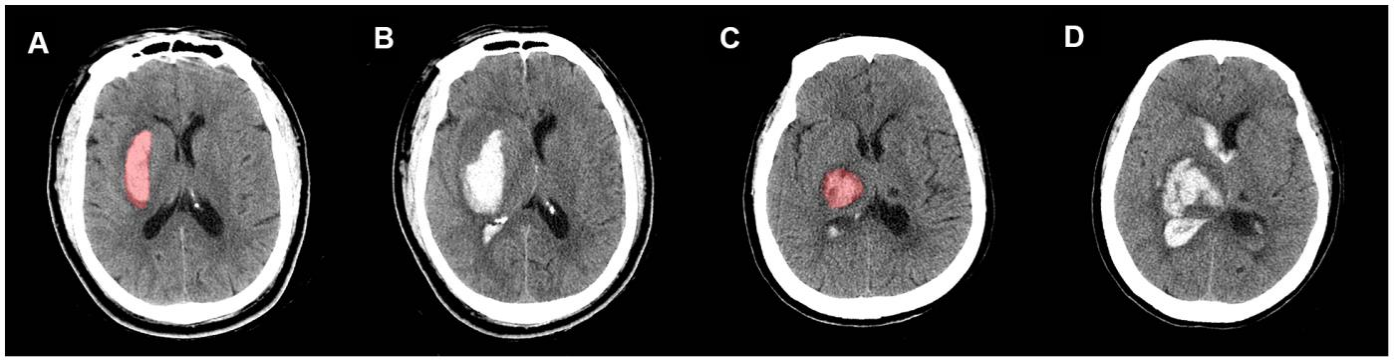
**Representative illustration of new IVH, IVH expansion, and manual region of interest (ROI) segmentation.** Images (**A**, **B**) are non-contrast CT images (axial view) of a 54-year-old male who experienced a new IVH. There was no baseline IVH (**A**), but the hematoma broke into ventricles on the follow-up CT (**B**). Images (**C**, **D**) are non-contrast CT images (axial view) of a 68-year-old female who experienced IVH expansion; (**C**) shows an initial IVH with a volume of 2.53 mL; follow-up CT (**D**) shows that the volume of IVH increased to 22.31 mL within 72 h.

New IVH refers to patients who had no baseline IVH but developed a new IVH lesion in follow-up CT images (<72 h) (Figure 5A, 5B). IVH expansion was defined as an absolute increase from the baseline IVH volume of >2 mL between the initial and follow-up CT images (Figure 5C, 5D). We chose a threshold of 2 mL to define IVH expansion because it is correlated with poor outcome and mortality [32]. IVH growth includes new and expanding IVH. We restricted hematoma to the deep brain region for several reasons. First, deep ICH locations are more likely to have HE [35]. Second, hematomas in the deep region (thalamus and basal ganglia) are closer to ventricle systems and have a higher risk of IVH growth and poor outcome [36, 37]. Third, in this exploratory study, we tried to exclude as many confounders as possible. Thus, we decided to exclude the non-deep hematomas and focus on the hematomas that had a greater risk for IVH growth (deep hematomas).

All CT images were acquired using a 64-channel multidetector CT scanner (LightSpeed VCT 64; GE Medical Systems, Milwaukee, WI, USA) with a scan thickness of 5 mm, reconstruction interval of 5 mm, tube voltage of 120 kV, tube current of 80 mAs, and matrix size of 512 × 512. Radiological data was acquired by two radiologists (two years of experience each) who were blinded to patients’ information. The baseline and follow-up IVH volume were measured using the “level tracing” function of 3D Slicer software (version 4.10.2; http://www.slicer.org). We also evaluated IVH severity by calculating the Graeb score [19] from the baseline and follow-up CT images. A Graeb score of ≥6 was defined as severe IVH based on the literature for IVH severity indicators [28, 33].

**Figure 6:**
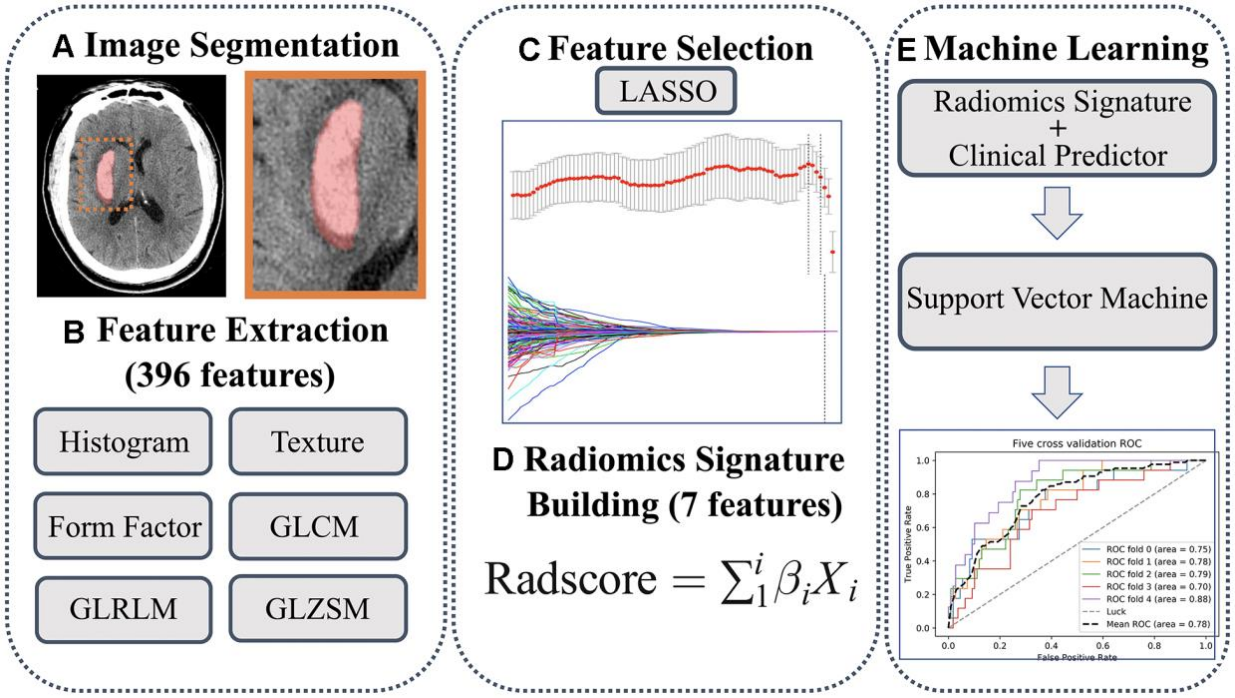
(**A**) Regions of interest were manually segmented. (**B**) A total of 396 features were extracted. (**C**) Features were selected using LASSO method. (**D**) Rad-score was calculated. (**E**) Predicting model was developed using support vector machine.

The segmentation process followed a consistent standard. Regions of interest (ROIs) were first manually segmented along the hematoma profile on each slice of non-enhanced CT (NECT) images (Figures 5, 6). Skull, peri-hemorrhagic edema, and normal brain parenchyma were manually excluded. All ROIs were first segmented by a radiologist with two years of experience. Then, to measure the inter-observer segmentation reproducibility, 100 images were randomly selected to be segmented by another radiologist with five years of experience [38, 39]. An inter-class correlation coefficient (ICC) of >0.75 was considered a good inter-observer agreement. A window width of 70 and a window level of 35 were set to clearly distinguished hematomas from brain parenchyma. All ROIs were examined by a senior radiologist with 10 years of experience.

A total of 396 radiomics features were extracted using the Artificial Intelligence Kit software (version 3.0.0.R; GE Healthcare). There were six categories: (1) Histogram, (2) Texture, (3) Form factor, (4) Grey level co-occurrence matrix (GLCM), (5) Grey level run-length matrix (GLRLM), and (6) Gray level size zone matrix (GLSZM) (more details about extracted features are shown in the Supplementary Table 1). To remove the unit data limits of each feature, the extracted features were standardized by z-score (Supplementary Material 1). The least absolute shrinkage and selection operator (LASSO) is suitable for feature selection in high-dimensional data [40]. Hence, we used the LASSO method to select the most valuable features. The parameter (λ) is a penalty parameter that varies for each model fitting step. As the value of λ increased, radiomics features with non-zero coefficients decreased. The optimal λ was selected by a 10-fold cross-validation, and the IVH growth-related radiomics features were subsequently chosen. The radiomics score (Rad-score) was calculated through the linear combination of selected features by multiplying with their respective LASSO coefficients. We categorized the Rad-score into groups by quartiles and cut-off value to explore the association between Rad-score, IVH severity, and clinical outcome. The workflow of ROI segmentation, feature extraction, and model construction is shown in Figure 6.

### Statistical analysis

Statistical analysis was performed with SPSS (version 24.0; IBM, Armonk, NY, USA) and R (version 3.6.1; http://www.R-project.org). Continuous variables are presented as medians (interquartile range, [IQR]), and categorical variables are shown as counts (with percentages). First, we performed univariate analysis to select potential clinical risk factors of IVH growth. Differences in continuous variables (age, time to initial CT, ICH volume, blood glucose, platelet count, international normalized ratio, Rad-score, and activated partial thromboplastin time) were examined using Student t-tests or Wilcoxon rank-sum test. The χ2 test or Fisher exact test (two-tailed) were performed for categorical variables (sex, GCS, history of hypertension, diabetes, hypercholesterolemia, hemorrhage, GOS, and alcohol consumption). A multivariable logistic regression with an enter method was performed to identify factors that were independently associated with IVH growth. Variance inflation factor (VIF) was used to detect multicollinearity, and a VIF of ≥5 was defined as multicollinearity [41]. Receiver-operator curve (ROC) analysis was conducted to derive the Rad-score cut-off value for IVH growth prediction, and the optimal cutoff value was selected using Youden’s index. A two-sided p value of <0.05 indicates a statistical difference.

### Machine learning and model performance evaluation

Support vector machine (SVM) is a type of supervised machine learning method that classifies data points by maximizing the margin between classes in a high-dimensional space [42]. The independent predictors identified in multivariable analysis were introduced into the SVM model. A five-fold cross-validation was used in the training cohort to determine the optimal hyperparameter, reduce overfitting, construct a stable model, and evaluate model performance [43]. The independent testing cohort was used to simulate the prediction and further test the model performance. (additional details about SVM modeling are shown in Supplementary Material 2). The machine learning process was performed using the scikit-learn packages (0.21.3) of Python (version 3.7; http://www.python.org).

The model performance was evaluated by the area under the receiver operating curve (AUC). Confusion matrix-derived metrics, including accuracy (ACC), sensitivity, specificity, true positive rate (TPR), true negative rate (TNR), positive predictive value (PPV), and negative predictive value (NPV), were also calculated.

### Evaluation of clinical usefulness

Decision curve analysis (DCA) measures the clinical utility of models by quantifying the net benefits at different threshold probabilities [44]. DCA was performed to compare the overall net benefits of the radiomics-clinical model and single clinical model without the Rad-score in the cohort. The net benefit was defined as the summation of benefits minus the holistic cost [45], and calculated using the formula: $\text{Net benefit}={\text{TPR}}_{\text{R}}\text{ P}-\frac{R}{1-R}\;FPR_{\text{R}}\;\;\left(1-P\right)$, where P is the disease prevalence; R is a threshold probability at which a patient will opt for treatment; TPR_R_ represents the proportion of cases with a model-calculated risk above threshold probability; and FPR_R_ represents the proportion of cases with a model-calculated risk lower than the threshold probability [45].

## Supplementary Material

Supplementary Materials

Supplementary Figures

Supplementary Tables
