# Supplementary material for: Predicting intraventricular hemorrhage growth with a machine learning-based, radiomics-clinical model
Source: Aging (Albany NY). 2021 May 4;13(9):12833–48. doi: 10.18632/aging.202954 (PMC8148477; doi:10.18632/aging.202954)
Supplement: Supplementary Materials [file aging-13-202954-s001.pdf]

SUPPLEMENTARY MATERIALS

Supplementary Materials 1

Z-score normalization

z-score normalization to make the image intensities have the properties of a standard normal distribution by scaling values to a mean of 0 and a standard deviation of 1 using the following formula:

$$z = \frac{\chi - \mu}{\sigma}$$

where μ was the mean value of the images, and σ was the standard deviation of images.

Supplementary Materials 2

Support vector machine model construction

We introduced five features, including history of hypercholesterolemia, baseline Graeb score, time to initial CT, INR and Rad-score, to the SVM model. Radial basis function (RBF) kernel function were applied and the optimal parameters of SVM were selected by grid-search method. Parameter C was 3.58e+01and parameter gamma was 6.87e-04. The 5-fold cross-validation was employed to validate the model performance in training cohort. The model was further validated in an independent testing cohort.

Supplementary Materials 3

Radiomics signature calculation formula

Rad-score = -
2.1378+0.135×Correlation_AllDirection_offset1_SD
-0.1723×Correlation_angle0_offset7
+0.5072×Correlation_angle0_offset4
-0.6258×HaralickCorrelation_AllDirection_offset4_SD
-0.1004×HaralickCorrelation_angle90_offset1
-0.353×ShortRunEmphasis_AllDirection_offset7_SD
+ 0.2577×ZonePercentage

Note: "SD" indicate the value reflects the standard deviation among the different directions.
