# Supplementary material for: Predicting intraventricular hemorrhage growth with a machine learning-based, radiomics-clinical model
Source: Aging (Albany NY). 2021 May 4;13(9):12833–48. doi: 10.18632/aging.202954 (PMC8148477; doi:10.18632/aging.202954)
Supplement: Supplementary Figures [file aging-13-202954-s002.pdf]

SUPPLEMENTARY FIGURES

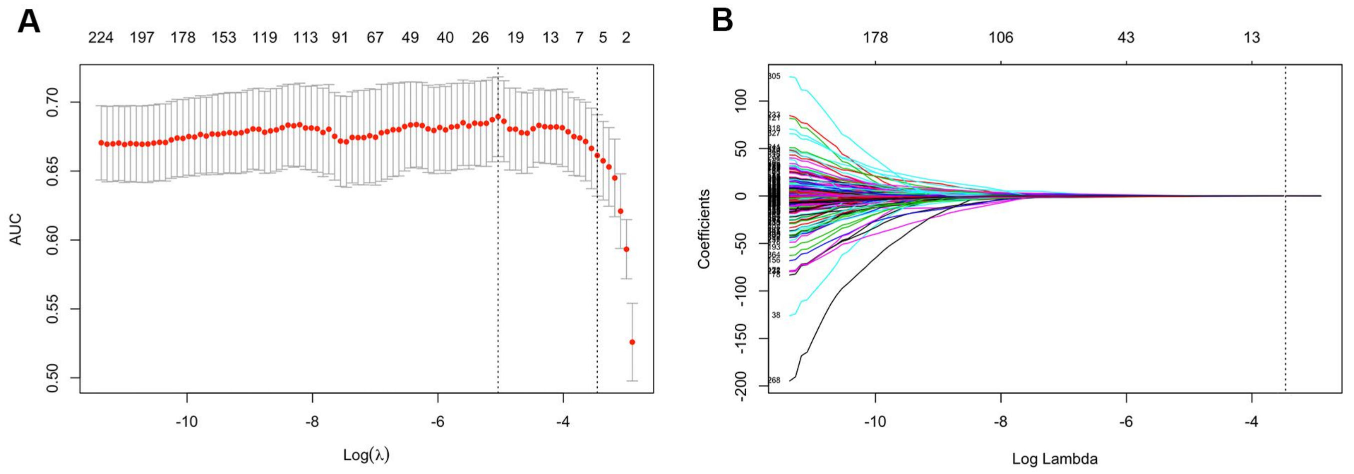

Supplementary Figure 1. Radiomics feature selection using the least absolute shrinkage and selection operator (LASSO) regression model. (**A**) Using 10-fold cross-validation to select tune parameter (λ). 7 features with non-zero coefficients were selected. Dotted lines on the left and right represent the minimum criterion and 1-standard error criterion (1-SE), respectively. The 1-SE criterion was applied in our study. A λ value of 0.03, with log (λ) of -3.46 was chosen (1-SE criteria). (**B**) LASSO coefficient profiles of the 396 radiomics features. The vertical line shows the optimal value of λ and 7 features with non-zero coefficients.

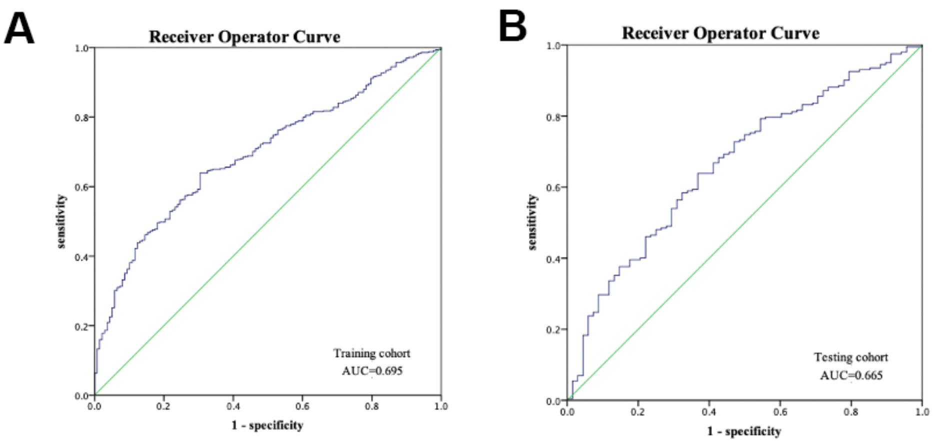

Supplementary Figure 2. Receiver operator curves (ROC) of Rad-score to predict poor out come (Glasgow Outcome Scale, ≤3) in training cohort (**A**) and testing cohort (**B**).
