# Supplementary material for: Predicting intraventricular hemorrhage growth with a machine learning-based, radiomics-clinical model
Source: Aging (Albany NY). 2021 May 4;13(9):12833–48. doi: 10.18632/aging.202954 (PMC8148477; doi:10.18632/aging.202954)
Supplement: Supplementary Tables [file aging-13-202954-s003.pdf]

SUPPLEMENTARY TABLES

Supplementary Table 1. Radiomics features extracted from regions of interest.

Group	Number	Feature
Histogram Parameters	42	Histogram parameters are concerned with properties of individual pixels. They describe the distribution of voxel intensities within the CT image through commonly used and basic metrics. Let *X* denote the three-dimensional image matrix with *N* voxels and *P* the first order histogram divided by *Nl* discrete intensity levels.
Texture Parameters	54	Texture is one of the important characteristics used in identifying objects or regions of interest in an image, texture represents the appearance of the surface and how its elements are distributed. It is considered an important concept in machine vision, in a sense it assists in predicting the feeling of the surface (e.g. smoothness, coarseness ...etc.) from image. Various texture analysis approaches tend to represent views of the examined textures form different perspectives.
Form Factor Parameters	9	These group of features includes descriptors of the three-dimensional size and shape of the tumor region.
GLCM Parameters	100	The Grey level co-occurrence matrix (GLCM) **P** (**I**, **j**|**Θ**, **d**) represents the joint probability of certain sets of pixels having certain grey-level values. It calculates how many times a pixel with grey-level i occurs jointly with another pixel having a grey value j. By varying the displacement vector d between each pair of pixels. The rotation angle of an offset: 0°, 45°, 90°, 135° and displacement vectors (distance to the neighbor pixel: 1, 2, 3 ...), different co-occurrence distributions from the same image of reference. GLCM of an image is computed using displacement vector d defined by its radius, (distance or count to the next adjacent neighbor preferably is equal to one) and rotational angles.
RLM Parameters	180	The grey level run-length matrix (RLM) **Pr**(**i**, **j** | **Θ**) is defined as the numbers of runs with pixels of gray level i and run length j for a given direction θ. RLMs is generated for each sample image segment having directions (0°,45°,90° and 135°), then the following ten statistical features were derived: short run emphasis, long run emphasis, grey level non- uniformity, run length non-uniformity, Low Grey Level Run Emphasis, High Grey Level Run Emphasis, Short Run Low Grey Level Emphasis, Short Run High Grey Level Emphasis, Long Run Low Grey Level Emphasis and Long Run High Grey Level Emphasis.
GLZSM Parameters	11	The gray level Size Zone Matrix (SZM) is the starting point of Thibault matrices. For a texture image f with N gray levels, it is denoted GSf(s, g) and provides a statistical representation by the estimation of a bivariate conditional probability density function of the image distribution values. It is calculated according to the pioneering Run Length Matrix principle: the value of the matrix GSf(s, g) is equal to the number of zones of size s and of gray level g. The resulting matrix has a fixed number of lines equal to N, the number of gray levels, and a dynamic number of columns, determined by the size of the largest zone as well as the size quantization. This matrix is particularly efficient to characterize the texture homogeneity, non periodicity or speckle like texture; it had provided betters characterizations than granulometry (or COM, RLM, etc.) for the classification of cell nuclei, dermis, road quality (bitumen condition) and some textures in PET images.

Supplementary Table 2. Multivariable analysis of features associated with IVH growth.

Variable	Odds ratio	95%CI	P value
Sex	-	-	0.116
Glasgow Coma Scale	-	-	0.131
Hypertension	-	-	0.076
Baseline IVH score	-	-	0.423
Baseline ICH volume	-	-	0.482
Blood glucose	-	-	0.987
PLT count	-	-	0.420
Hypercholesterolemia	0.12	0.02-0.90	0.039
INR	4.27	1.40-13.0	0.011
Baseline Graeb score	1.26	1.16-1.36	<0.001
Time to initial CT	0.70	0.58-0.86	<0.001
Rad-score	2.3	1.6-3.3	<0.001

Abbreviations: ICH=intracerebral hemorrhage; IVH=intraventricular hemorrhage; INR= international normalized ratio.

Supplementary Table 3. Details about the selected features.

Feature name	ICC	Group
Correlation_AllDirection_offset1_SD	0.91	Texture Parameters
Correlation_angle0_offset7	0.89	Texture Parameters
Correlation_angle0_offset4	0.90	Texture Parameters
HaralickCorrelation_AllDirection_offset4_SD	0.81	GLCM
HaralickCorrelation_angle90_offset1	0.90	GLCM
ShortRunEmphasis_AllDirection_offset7_SD	0.80	GLRLM
Zone Percentage	0.98	GLZSM

Abbreviations: GLCM= Grey level co-occurrence matrix, GLRLM= Grey level run-length matrix, GLSZM= Gray level size zone matrix, ICC= Inter-class correlation coefficient.

Supplementary Table 4. Confusion matrices of training cohort.

Training cohort (N=626)		Actual	
		Positive	Negative
Predicted	Positive	70	186
	Negative	14	356

Supplementary Table 5. Confusion matrices of testing cohort.

Testing cohort (N=270)		Actual	
		Positive	Negative
Predicted	Positive	30	85
	Negative	7	148
